# Diabetes and the COVID-19 pandemic

**DOI:** 10.1007/s00125-022-05833-z

**Published:** 2022-11-23

**Authors:** Kamlesh Khunti, Jonathan Valabhji, Shivani Misra

**Affiliations:** 1 Diabetes Research Centre, University of Leicester, Leicester, UK; 2 Division of Metabolism, Digestion & Reproduction, Imperial College London, London, UK; 3 Diabetes & Endocrinology, Imperial College Healthcare NHS Trust, London, UK

**Keywords:** Coronavirus, COVID-19, Diabetes, Pandemic, Review

## Abstract

Almost immediately after the severe acute respiratory syndrome coronavirus 2 (SARS-CoV-2) virus emerged, it was evident that people with chronic diseases, including diabetes, were disproportionately affected, with an increased risk of hospitalisation and mortality. Over the ensuing 2 years, the indirect effects of the pandemic on healthcare delivery in the short term have become prominent, along with the lingering effects of the virus in those directly infected. In the wake of the pandemic and without any evidence from high quality studies, a number of national and international consensus recommendations were published, which were subsequently rapidly updated based on observational studies. There have been unprecedented disruptions from both direct and indirect impacts of coronavirus disease-2019 (COVID-19) in people with diabetes. In this review, we summarise the impact of acute COVID-19 in people with diabetes, discuss how the presentation and epidemiology during the pandemic, including presentation of diabetic ketoacidosis and new-onset diabetes, has changed, and we consider the wider impact of the pandemic on patients and healthcare service delivery, including some of the areas of uncertainty. Finally, we make recommendations on prioritising patients as we move into the recovery phase and also how we protect people with diabetes for the future, as COVID-19 is likely to become endemic.

**Graphical abstract:**

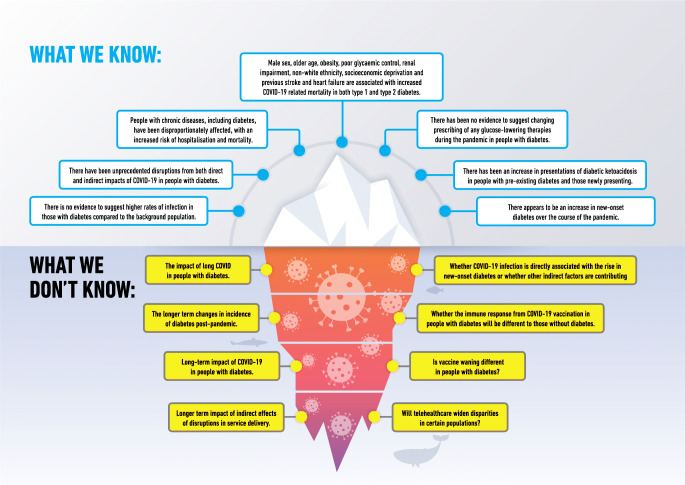

**Supplementary Information:**

The online version contains a slideset of the figures for download, which is available to authorised users at 10.1007/s00125-022-05833-z.

## Introduction

Almost immediately after the severe acute respiratory syndrome coronavirus 2 (SARS-CoV-2) virus emerged, it was evident that diabetes was a key player in determining outcomes for those affected. Over the ensuing 2 years, the indirect effects of the pandemic on healthcare delivery in the short term have become prominent, along with the lingering effects of the virus in those who have been infected. This first global pandemic of the modern age has unleashed the full potential of the scientific community, and in vitro studies, human trials and big-data analyses have all been utilised to help decipher the complex interactions between coronavirus disease-2019 (COVID-19) and diabetes. This review discusses the current literature to answer three fundamental questions: (1) what is the impact of acute COVID-19 in people with diabetes; (2) how has the presentation and epidemiology of new-onset diabetes changed during the pandemic; and (3) what is the wider impact of the pandemic on patients and healthcare service delivery?

## Acute COVID-19 in people with diabetes

The early reports from Wuhan, China suggested an over-representation of people with diabetes in those dying from COVID-19 [1]. The first report suggesting an independent effect of diabetes status on COVID-19-related mortality came from English data, studying records of over 17 million adults linked to 10,926 COVID-19-related deaths [2]. The fully adjusted hazard ratio (HR) for those with diabetes and HbA_1c_<58mmol/mol (<7.5%) was 1.31 (95% CI 1.24, 1.37), and for those with HbA_1c_≥58mmol/mol (≥7.5%) was 1.95 (1.83, 2.08). A subsequent larger study assessing the entire English population reported the odds ratios (OR) of in-hospital COVID-19-related mortality, by type of diabetes. Records of 263,830 (0.4%) individuals with a recorded diagnosis of type 1 diabetes, 2,864,670 (4.7%) individuals with a diagnosis of type 2 diabetes, 41,750 (0.1%) individuals with other types of diabetes and 58,244,220 (94.8%) individuals with no diabetes were linked to 23,698 in-hospital COVID-19-related deaths [3]. Adjusted for age, sex, deprivation, ethnicity and geographical region, compared with people without diabetes, the OR for in-hospital COVID-19-related death was 3.51 (95% CI 3.16, 3.90) in people with type 1 diabetes and 2.03 (1.97, 2.09) in people with type 2 diabetes. The relative effect of having diabetes was greater in younger people: in those younger than 70 years, compared with people with no diabetes, the OR was 6.39 (95% CI 5.40, 7.56) for people with type 1 diabetes and 3.74 (3.50, 3.99) for those with type 2 diabetes. The corresponding ORs for those older than 70 years were 2.81 (2.46, 3.22) and 1.79 (1.74, 1.85), respectively.

Using national diabetes data in England, Holman et al reported associations between risk factors and COVID-19-related mortality in 264,390 people with type 1 diabetes and 2,874,020 people with type 2 diabetes [4]. Male sex, older age, renal impairment, non-white ethnicity, socioeconomic deprivation and previous stroke and heart failure were associated with increased COVID-19-related mortality in both type 1 and type 2 diabetes. Compared with people with an HbA_1c_ of 48–53 mmol/mol (6.5–7.0%), people with an HbA_1c_ of ≥86 mmol/mol (≥10%) had increased COVID-19-related mortality (HR 2.23 [95% CI 1.50, 3.30] in type 1 diabetes and 1.61 [1.47, 1.77] in type 2 diabetes). The association between BMI and COVID-19-related mortality was U-shaped: in type 1 diabetes, compared with people with a BMI of 25.0–29.9 kg/m^2^, people with a BMI of less than 20.0 kg/m^2^ had an HR of 2.45 (95% CI 1.60, 3.75) and those with a BMI of 40.0 kg/m^2^ or higher had an HR of 2.33 (1.53, 3.56). The corresponding HRs for type 2 diabetes were 2.33 (2.11, 2.56) and 1.60 (1.47, 1.75). Subsequent reports from whole populations, first from Scotland [5], then from Sweden [6], confirmed similar signals.

A systematic review of 18 studies reported a pooled prevalence of diabetes of 11.5% (95% CI 9.7, 13.4), with diabetes being associated with a high risk of severe COVID-19 (relative risk [RR] 2.11; 1.40, 3.19) compared with those without [7]. Despite the higher COVID-19-related risks for hospitalisation, intensive care unit admission and mortality for those with diabetes, there has been no evidence to suggest higher rates of infection in those with diabetes [8].

## International guidelines for management of people with diabetes during the pandemic

When it became clear that people with diabetes were disproportionately affected in terms of hospitalisation and mortality, and in the absence of evidence to inform clinical practice, a number of consensus recommendations were published [9, 10]. Many country-specific guideline recommendations included outpatient and inpatient management, urgent and acute diabetes care, foot services, pregnancy services, management in emergency departments, therapeutic targets and routine use of glucose-lowering medications in clinical practice [10, 11]. Guidance for specific populations, such as those fasting for Ramadan during COVID-19 pandemic, were also published [12]. There were subsequent updates on practical recommendations, including post-pandemic recommendations [13].

### Glucose-lowering therapies for people with diabetes during the pandemic

Most guidelines recommended tight glycaemic control in people with diabetes, more intense monitoring, and supporting therapies to reduce the risk of diabetic ketoacidosis (DKA) [9, 11, 13]. In particular, caution was recommended regarding the use of sodium–glucose cotransporter 2 (SGLT2) inhibitors due to concerns around ketoacidosis [9, 11]. Consistent findings from observational studies of people admitted in hospital reported that use of metformin was associated with better outcomes, whereas insulin was associated with worse outcomes. A meta-analysis of 18 studies with 12,277 patients with diabetes and COVID-19 reported that insulin was significantly associated with increased risk of mortality [14]. Interestingly, insulin treatment was associated with a significantly increased risk of hospital admission in people with type 2 diabetes (OR 1.31; 95% CI, 1.06, 1.61) but a lower risk in people with type 1 diabetes (OR 0.14; 0.05, 0.35), although these results were based on a limited number of studies [14].

In contrast, a meta-analysis of nine studies with 10,233 people with type 2 diabetes reported lower mortality risk in people with diabetes on metformin (OR 0.64; 95% CI 0.43, 0.97) [15]. Another systematic review of 31 studies and 66,914 patients reported that metformin was associated with significantly lower mortality risk (OR 0.62; 95% CI 0.50, 0.76), slightly lower mortality for sulfonylureas/glinides (OR 0.93; 0.89, 0.98) and dipeptidyl-peptidase 4 (DPP4) inhibitors were associated with a statistically non-significant lower mortality risk (OR 0.95; 0.72, 1.26) [16]. A further systematic review and meta-analysis of ten studies with 7012 COVID-19 patients with diabetes reported that there was no association between DPP4 inhibitors and severe COVID-19 (OR 1.07; 95% CI 0.87, 1.31) or mortality (OR 1.14; 0.87, 1.51) [17]. Another meta-analysis of nine studies with 19,660 people with diabetes with COVID-19 suggested that pre-admission use of glucagon-like peptide-1 (GLP-1) receptor agonists was associated with reduced mortality (OR 0.53; 95% CI 0.43, 0.66) [18].

The largest study included over 2.5 million people with type 2 diabetes in a nationwide observational study in England [19]. Overall the study findings showed that the adjusted HR for mortality for metformin was 0.77 (95% CI 0.73, 0.81), 1.42 (1.35, 1.49) for insulin, 0.82 (0.74, 0.9) for SGLT2 inhibitors, 0.94 (0.82, 1.07) for thiazolidinedione, 0.94 (0.89, 0.99) for sulfonylureas, 0.94 (0.83, 1.07) for GLP-1 receptor agonists and 1.07 (1.01, 1.13) for DPP4 inhibitors. However, the absolute differences in the risk were small and these findings were likely to be due to confounding by indication. The study recommended that there was no clear indication to change prescribing of glucose-lowering therapies during the pandemic in people with type 2 diabetes [19].

All observational studies have a number of sources of bias, including individual behaviours, individual characteristics, healthy user and adherer effects and confounding by selective prescribing [20]. RCTs are regarded as the gold standard for evaluating effectiveness of interventions, including pharmacological interventions. However, there have been few RCTs of glucose-lowering therapies in people with diabetes who have had COVID-19. One such study is the dapagliflozin in respiratory failure in patients with COVID-19 (DARE-19) trial, which was a double-blind, placebo-controlled RCT of 1250 patients, with and without diabetes, who were hospitalised with COVID-19 and who had at least one cardiovascular risk factor [21]. This study showed dapagliflozin was well tolerated and numerically resulted in fewer events of organ dysfunction but no statistically significant difference in the dual primary outcome of prevention (time to new or worsening organ dysfunction or death), or the hierarchical composite outcome of recovery by 30 days.

### Identifying people with diabetes at high risk for population-level interventions

Over the course of the pandemic, a number of prediction models for severe manifestations and mortality due to COVID-19 have been derived to inform decisions about shielding, mitigating occupational exposure and prioritising for COVID-19 vaccinations. A recent systematic review highlighted two models as the most promising and well validated [22]. The first applied the Qresearch database, comprising 1205 general practices with 8.25 million adults aged 19–100 years in England, and used clinical data derived from individuals from before the pandemic to explore risks of COVID-19-related mortality. The final risk algorithms included age, ethnicity, deprivation, BMI and a range of comorbidities and performed well. For women and men with type 1 diabetes, adjusted HRs of COVID-19-related mortality at mean age were 4.02 (95% CI 2.07, 7.82) and 5.84 (3.97, 8.60), respectively, and for women and men with type 2 diabetes, adjusted HRs of COVID-19-related mortality at mean age were 6.29 (4.08, 9.70) and 4.74 (3.34, 6.71), respectively [23].

The second study derived a risk score to predict mortality in patients admitted to hospital with COVID-19. In total, 35,463 patients were included in the derivation dataset (mortality rate 32.2%) and 22,361 in the validation dataset (mortality rate 30.1%) using admission data. The final 4C Mortality Score included eight variables readily available at initial hospital assessment (age, sex, number of comorbidities [including diabetes], respiratory rate, peripheral oxygen saturation, level of consciousness, urea level and C reactive protein) and showed high discrimination for mortality with excellent calibration [24].

Subsequently, the Qresearch database was again applied to derive and validate risk prediction algorithms to estimate the risk of COVID-19-related mortality and hospital admission in UK adults after one or two doses of COVID-19 vaccination. The adjusted HR for COVID-19-related mortality for those with type 2 diabetes and HbA_1c_ less than 59 mmol/mol (<7.5%) was 1.26 (95% CI 1.12, 1.42), and for those with HbA_1c_ greater than or equal to 59 mmol/mol (≥7.5%) was 1.43 (1.21, 1.70). However, absolute risks for all groups were significantly lower following vaccination. There was insufficient power to assess risks for those with type 1 diabetes following vaccination [25].

## How has the presentation and epidemiology of new-onset diabetes and DKA changed during the pandemic?

### The direct and indirect impacts of COVID-19 on diabetes

Shortly after the pandemic began, reports emerged of an increase in presentations of new-onset diabetes [26–29], as well as DKA in people with pre-existing diabetes and in those with new-onset diabetes [27, 28, 30–33]. Many hypothesised a bi-directional relationship between COVID-19 and diabetes [34–36], whereby having diabetes was itself a risk factor for worse outcomes from COVID-19, but also, having COVID-19 was a risk factor for newly diagnosed diabetes and/or hyperglycaemic emergencies.

### DKA during the pandemic

One of the first national studies comparing DKA to preceding years came from the German Diabetes Prospective Follow-up Registry of children and adolescents presenting with type 1 diabetes [37]. In 532 children with newly diagnosed type 1 diabetes presenting over the 3 months of the first wave, the frequency of DKA at presentation was significantly higher (44.7% vs 24.5% in 2019 vs 24.1% in 2018), as was the proportion presenting with severe DKA. The adjusted RR for DKA was highest in children under 6 years, females and those from an immigrant background. More granularity emerged from a population-wide study in England [38] analysing trends in DKA admissions across two waves of the pandemic and the period between waves in those with pre-existing type 1, type 2 and newly diagnosed diabetes. In people with type 1 diabetes, DKA admissions were significantly reduced across all study periods, but to a lesser extent between the waves. This was most pronounced in children and young adults under the age of 40 years (in whom reductions in recurrent admissions was also observed). For those with pre-existing type 2 diabetes, DKA admission rose significantly across all periods. In those newly diagnosed with diabetes at the time of their admission, rates of DKA were higher compared with preceding years across all the pandemic periods studied; 57% higher during the first wave, 56% higher in between waves (when rates of COVID-19 positivity were almost zero) and 61% higher during the second wave. Unlike the age associations observed for the reduction in DKA in those with type 1 diabetes (younger age groups) and increase in type 2 diabetes (older age groups), the high rates of DKA in those newly diagnosed was observed across all age categories.

In a more ethnically diverse US study [39] (>30% of 15,000 people studied were black, Hispanic or Asian), a rise in DKA cases in those with pre-existing type 1 diabetes was found during the first two COVID-19 waves compared with matched time periods in 2019, and the rise was more pronounced in non-Hispanic black individuals compared with non-Hispanic white.

Whilst most studies have shown a rise in DKA incidence in those newly presenting with diabetes (type 1 or other) the findings in those with pre-existing type 1 diabetes are less clear, with the English study of national data showing a reduction in incidence during COVID waves [38], whilst the US study showed an increase [39]. One reason for this may be the small numbers of non-white individuals in the English study inadequately capturing trends in these ethnic groups, along with differences in the way DKA and type 1 diabetes was defined. However, variation in DKA incidence between countries and geographies is well documented, and other healthcare factors and social determinants may also account for these differences [40].

The reduction of DKA in people with type 1 diabetes and increase in people with type 2 diabetes is supported by studies examining glycaemic control, which showed no deterioration of control in those with type 1 diabetes during the pandemic, in contrast to those with type 2 diabetes where a rise in HbA_1c_ and BMI was noted [41, 42].

### Putative explanations for excess DKA

DKA is a largely preventable complication in individuals with pre-existing type 1 diabetes, where infection or insulin omission are the primary causes [43], and in those newly presenting with type 1 diabetes, with earlier diagnosis through greater recognition of the initial symptoms. However, DKA may also occur in type 2 diabetes during catabolic illness, in which an excess of counterregulatory hormones can precipitate a relative insulin deficiency, and may occur as a hybrid presentation with hyperglycaemic hyperosmolar state (a more common diabetic emergency in people with type 2 diabetes).

The reduction of DKA in people with pre-existing type 1 diabetes observed in some studies may be accounted for by the considerable shifts in lifestyle imposed by lockdown, and it is possible that in those usually vulnerable to DKA these changes mitigated DKA. For those with type 2 diabetes, the trend observed is interesting as the demographic most affected are older individuals, men and those from minority ethnic groups. These are also the characteristics of people most likely to have severe outcomes from COVID-19 [4]. One may hypothesise that the higher levels of DKA during the peak of the COVID-19 pandemic may coincide with COVID-19 infection and represent the development of ketoacidosis in critically unwell individuals [44, 45]. It is not possible from existing studies to ascertain whether people were admitted because of DKA or whether DKA was coincidental at the time of admission with COVID-19, or even whether it developed after admission. However, this cannot be the only explanation as the rate of DKA remained higher in between pandemic waves. It could be speculated that DKA occurred in an at-risk type 2 diabetes population who did not have the tools to intensify control; analyses have shown high HbA_1c_ levels in those presenting with DKA and type 2 diabetes [44], with only 38% of people insulin-treated.

The rise of DKA observed in people with newly diagnosed diabetes and in those with pre-existing type 2 diabetes suggests factors other than acute SARS-CoV-2 infection may contribute to the development of DKA. These include changes in lifestyle that may have led to decompensation of type 2 diabetes control, including significant weight gain [46], a reduction in contact with healthcare providers to discern deteriorating control (see next section), a trend in line with the year-on-year rise in DKA [47], or indeed a downstream effect of previous COVID-19 infection.

### New-onset diabetes during the pandemic

Multiple studies have indicated a higher incidence of new-onset diabetes during the pandemic [48]. One large cohort study, which used the national database of the US Department of Veterans Affairs to build a cohort of 181,280 participants with a positive COVID-19 test, showed that, at a median follow-up of 352 days, people with COVID-19 had an increased risk of diabetes compared with the control group (OR 1.40; 95% CI 1.36, 1.44) [49].

The precise mechanism for new-onset diabetes in COVID-19 is not yet known but may include previously undiagnosed diabetes, stress hyperglycaemia, steroid-induced hyperglycaemia or direct or indirect effects of SARS-CoV-2 infection on the pancreatic beta cells [48]. One recent cross-sectional population-based study observed a slightly higher, but non-significant, increase in diabetes incidence in children in Canada during the pandemic [50]. The authors suggested that this could be due to delays in diagnosis during the early stages of the pandemic, with catch-up effect. This rise in new-onset diabetes has raised the possibility of a SARS-CoV-2 specific effect on the beta cells of the pancreas, compromising insulin production [51]. This hypothesis has been fuelled by multiple reports of new diabetes in children and reports of excess DKA in newly diagnosed individuals, or indeed insulin-requiring hyperglycaemia [52, 53]. If proven, SARS-CoV-2 would be the first such virus to target pancreatic beta cells in this way.

**Fig. 1:**
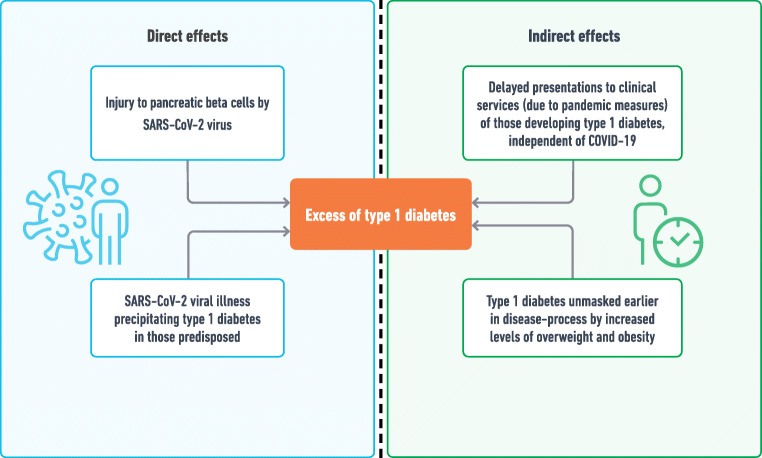
Potential mechanisms by which COVID-19 could lead to an increase in type 1 diabetes. This figure is available as part of a downloadable slideset

New-onset diabetes can be challenging to classify in patients presenting with DKA, especially in adults from non-European ethnicities; type 1 diabetes cannot always be assumed, instead DKA could represent ketosis-prone type 2 diabetes or indeed bona fide type 2 diabetes with a catabolic presentation. However, in children, the prior probability of type 1 diabetes is much higher. Although autoimmune in pathogenesis, the precipitation of type 1 diabetes usually requires a ‘second hit’ or environmental trigger, including infection [54, 55]. Hypothetically, there are several mechanisms by which the SARS-CoV-2 virus could directly lead to an increase in type 1 diabetes (Fig. 1): (1) the virus itself could directly injure pancreatic beta cells, which could occur whether a person is predisposed to autoimmune disease or not [51, 56]; (2) SARS-Cov-2 infection could be the trigger or ‘second hit’ precipitating type 1 diabetes in those predisposed; and (3) COVID-19 infection could simply unmask hyperglycaemia earlier in the disease trajectory in those already developing type 1 diabetes. Disentangling these potential ‘direct effects’ of the SARS-CoV-2 virus from ‘indirect effects’ is hugely challenging and studies analysing these associations have been complicated by confounders (Text Box 1). Indirect effects of the pandemic include a rise in levels of overweight and obesity due to lockdown measures, which could hasten the presentation of any type of diabetes [46], and changes in exposure of other endemic viruses [57, 58] that may have altered immune responses in those susceptible to developing type 1 diabetes. 
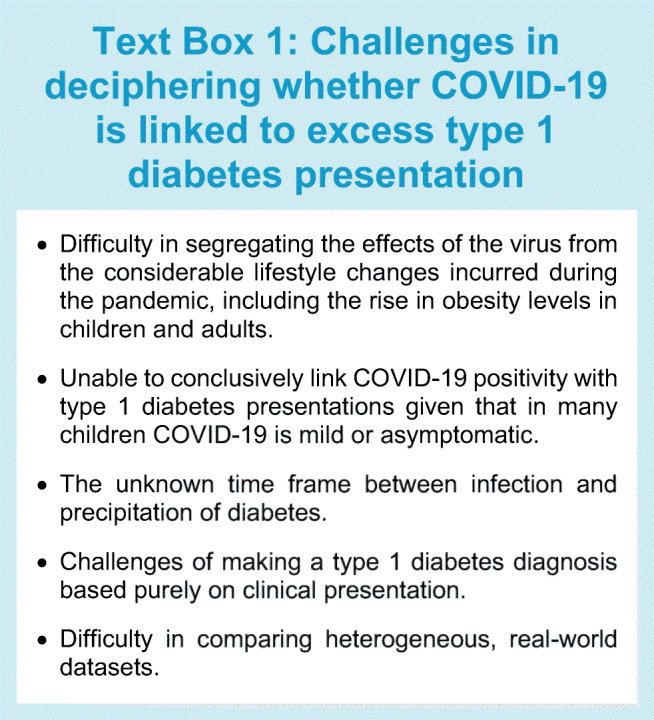

Whether new-onset diabetes following SARS-CoV-2 infection is likely to remain permanent is not known as there are limited data on follow-up of patients newly diagnosed with COVID-19. In a study of 1902 people admitted with COVID-19, 31.2% had pre-existing diabetes and 13% had new-onset diabetes, which was more common in younger patients and less common in those of non-Hispanic white ethnicity [59]. Out of the survivors with new-onset diabetes, 56.3% continued to have diabetes and 40.6% regressed to normoglycaemia or prediabetes, and two were unable to be classified at a median follow-up of 323 days [59].

## What is the wider impact of the pandemic on patients and healthcare service delivery?

### Long COVID

As well as the acute effects of COVID-19, many survivors are now reporting incomplete recovery many months after acute infection, which has been referred to as long COVID. In December 2020, the National Institute for Health and Care Excellence (NICE) guidelines in the UK defined long COVID as the persistence of symptoms beyond 4 weeks of SARS-CoV-2 infection [60], including both ongoing symptoms occurring between 4 and 12 weeks and the post-COVID-19 syndrome when symptoms continue beyond 12 weeks. Recently, the WHO published a new definition of post-COVID-19 condition which refers to it as the persistence of symptoms 3 months after SARS-CoV-2 infection, lasting for at least 2 months and not explained by other illnesses [61]. The most common symptoms of long COVID include fatigue and cough. Globally, long COVID is a major research focus [62], although there is little data on long COVID specifically in people with diabetes. One small case–control study reported that the most prevalent post-COVID symptoms were fatigue, breathlessness on exertion, and pain, but that there were no differences in limitations in activities of daily living nor differences in symptoms between those with and without diabetes [63]. Recently, a large US Department of Veterans Affairs cohort study of 153,760 individuals with COVID-19 showed that, beyond the first 30 days after infection, the overall risk of incident CVDs (including cerebrovascular disorders, dysrhythmias, ischaemic heart disease, pericarditis, thrombotic disorders, major adverse cardiovascular events and any cardiovascular outcomes) was increased in both people with and without diabetes, although the HR was lower in individuals without diabetes compared with people with diabetes [64].

### COVID-19 effects on service delivery

In most nations, healthcare delivery outside of the acute setting was severely compromised as part of the pandemic response. This usually included a marked reduction of community/general practice services, restrictions to face-to-face consultations in both primary and secondary care, a shift to virtual consultations and omission of other facets of the consultation such as body measurements, blood tests and annual diabetes reviews [65, 66].

Whilst the direct risks of COVID-19 in people with diabetes have been well reported, data on the indirect effects of COVID-19 due to disruption in care have been lacking. One study assessed 25 million patients in the UK from general practice records and reported a significant reduction in type 2 diabetes diagnoses during the first wave of the pandemic (rate reduction in new diagnoses of 0.7) [66]. Although there was some recovery, the rate remained reduced later in 2020, and they estimated missed or delayed diagnoses of type 2 diabetes in ~60,000 individuals. One may speculate that a proportion of these will account for the excess of DKA presentations in ‘newly diagnosed’ diabetes categories.

In most nations, there are standards of care for people living with diabetes. For example, in England, people with diabetes should receive nine annual care processes. Delivery of care processes are important as higher completion has been associated with reduced mortality in a number of studies [67, 68]. In one pandemic study [69], the rates of performing these checks in people with type 2 diabetes reduced by 76–88% when compared with 10-year trends, disproportionately affecting older people from deprived areas. It is important to segregate immediate effects of a reduction in care delivered and the immediate effects of lockdown on outcomes, from the longer-term effects of this acute reduction in routine care delivery to people with diabetes. Delivery of care processes has also been associated with a longer-term reduction in lower limb amputation [70]. An English population study during the first wave of the pandemic [71] demonstrated significant reductions in rates of lower limb major amputations, minor amputations and revascularisation procedures in those with diabetes, compared with previous years.

A recent population-based parallel cohort study over a 15 week period in 2021 in England found that reduced delivery of eight care processes (checks of HbA_1c_, blood pressure, cholesterol, serum creatinine, urine albumin, foot surveillance, BMI and smoking status) following the pandemic onset was associated with a higher non-COVID-19-related mortality [72]. The incident rate ratio for non-COVID-19-related mortality, compared with the all-cause mortality over the same period in 2019 after adjustment for age, sex, ethnicity, deprivation and diabetes type, was 1.02 (95% CI 1.01, 1.04). Non-COVID-19-related mortality in 2021 was highest in people who did not receive all eight care processes in either of the two previous years (OR 2.67; 95% CI 2.56, 2.77), and intermediate in those who received all eight care processes in one of the two previous years, compared with those who received all eight care processes in both previous years [72].

## Recovery from the pandemic

### Inpatient setting

Potential reasons for deterioration of glycaemic control in people admitted to hospital include acute or chronic inflammation leading to worsening of insulin resistance and hyperglycaemia. Inpatient management should include delivery of care by multidisciplinary diabetes teams, use of technology including continuous glucose monitoring, achieving good glycaemic control, use of evidence-based therapies for COVID-19, improving patient education and also ensuring well-being of healthcare workers [73–75]. In view of the high risk of mortality or readmission, early routine follow-up would be important for patients discharged following admission due to SARS-CoV-2 infection [73].

### Community setting

**Fig. 2:**
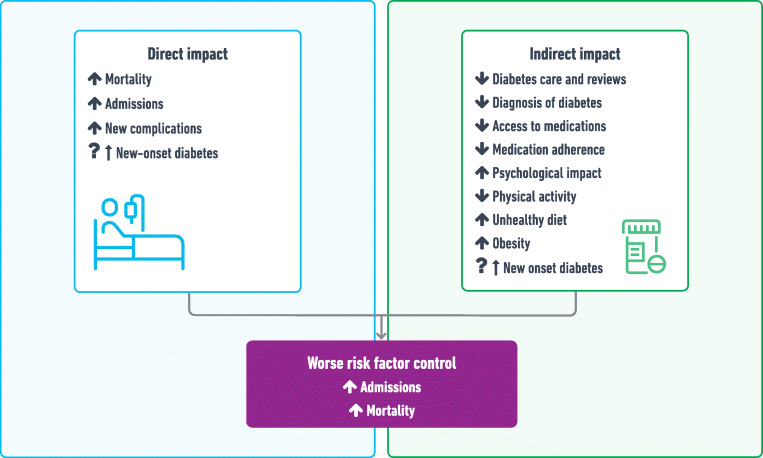
Direct and indirect impacts of COVID-19 on people with diabetes. This figure is available as part of a downloadable slideset

Previous natural disasters have shown that short-term disruptions to healthcare are associated with worse outcomes for people with diabetes within the short to medium term, including poor control of intermediate risk factors, such as glycaemic control, blood pressure, lipid control and worsening of mental health, and an overall negative impact on health and the economy [76–79]. The direct and indirect impact over 2 years of disruptions due to the pandemic will take time to manifest but are likely to be worse than the direct impact COVID-19 has had on people with diabetes (Fig. 2).

The COVID-19 pandemic has posed significant healthcare challenges for both healthcare professionals and patients [80]. As we now go into the recovery phase, it is imperative that people with diabetes are prioritised for early assessment of the care processes, including face-to-face consultations. Many healthcare organisations had been considering remote consultations (a key part of the pandemic response including telephone, videos and e-consultations), but the COVID-19 public health emergency has accelerated this initiative [81].

It is important to ensure that the inequalities seen during COVID-19 are not exacerbated with remote consultations, particularly for those who have difficulty with access to such consultations, including deprived populations, older adults and ethnic minority populations [82].

A number of organisations have suggested guidance for risk stratification to prioritise diabetes reviews for those at highest risk [83, 84]. However, simple pragmatic risk-based reviews could include people who are already known as being at highest risk, including people with poor pre-pandemic risk factor control, those living with obesity, those with microvascular complications (particularly chronic kidney disease), those with CVD and those with multiple comorbidities.

As well as prioritising patients who have not been reviewed during the pandemic, those who have been directly affected by COVID-19 will need to be followed up carefully. An Italian study reported that people aged between 45 and 94 years who tested positive for SARS-Cov-2 and were hospitalised had a significantly higher all-cause mortality (incidence rate ratio [IRR] 1.92; 95% CI 1.63, 2.25), and diabetes was associated with an increased risk of first cardiovascular event hospitalisation (IRR 2.24; 2.18, 4.25) [85]. In another study from England of people with COVID-19 who were discharged from hospital, 12.3% died and 29.4% were readmitted within 140 days with an IRR 3.5 (95% CI 3.4, 3.6) and 7.7 (7.2, 8.30) compared with the matched control participants [86]. The rates of respiratory disease, diabetes and new cardiovascular events were also significantly increased in patients discharged following COVID-19 [86]. It is therefore clear that people who have tested positive for COVID-19 or who have been admitted with COVID-19 will need close follow-up in the short to medium term.

Studies have shown that poor glycaemic control is associated with the worst outcomes, including mortality [19], and improving risk factor control is a priority as we open up to routine clinical care following lockdowns. There have been recent contradictory studies reporting the association of hyperglycaemia with vaccine effectiveness. One large study observed that COVID-19 vaccination induced a weak immunity in people with type 2 diabetes and poor glycaemic control, compared with those with normoglycaemia [87]. Another study of 161 participants with type 1 and type 2 diabetes and 86 healthy control individuals observed that anti-SARS-CoV-2 receptor-binding antibody levels were comparable in healthy individuals and participants with type 1 and type 2 diabetes irrespective of glycaemic control [88]. Another recent Italian study found that poor glycaemic control was associated with an increased risk of COVID-19 breakthrough infections in people with diabetes [89]. The study concluded that poor glycaemic control after vaccination may worsen the immunological response to vaccines, which might favour SARS-CoV-2 infections in people with diabetes. Despite these contrasting findings, it is important to keep individualised glycaemic goals and prioritise these patients for vaccination.

## Summary

Throughout the SARS-CoV-2 pandemic, people with chronic diseases, including diabetes, have been disproportionately affected, with an increased risk of hospitalisation and mortality. Both direct and indirect effects of COVID-19 have had a substantial impact on people with diabetes, and whilst much has been learnt from this, there remain many areas of uncertainty (Text Box 2). As we move into the recovery phase, prioritising routine clinical reviews for risk factor control, appropriate therapeutic management and addressing mental well-being through self-management educational programmes is paramount. In view of the many uncertainties regarding the direct and indirect effects of COVID-19, people with diabetes will need to be followed up closely. Without this close oversight, there is likely to be a greater risk of poor outcomes in the short to medium term, and with it a significant burden to the healthcare system. 
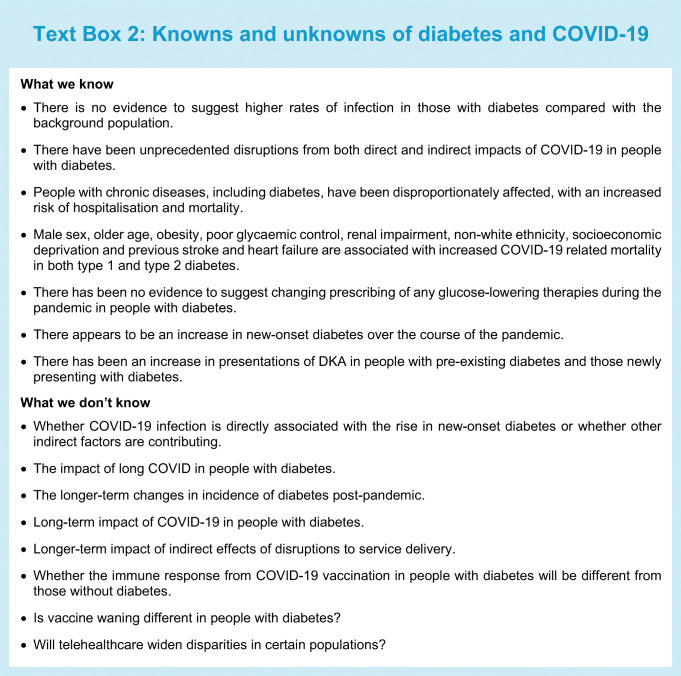


## Supplementary Information

Slideset of figures (PPTX 212 kb)

## Abbreviations

COVID-19: Coronavirus disease-2019
DKA: Diabetic ketoacidosis
DPP4: Dipeptidyl-peptidase 4
GLP-1: Glucagon-like peptide-1
HR: Hazard ratio
IRR: Incidence rate ratio
OR: Odds ratio
RR: Relative risk
SARS-CoV-2: Severe acute respiratory syndrome coronavirus 2
SGLT2: Sodium–glucose cotransporter 2
