# Supplementary material for: Diabetes and the COVID-19 pandemic
Source: Diabetologia. 2022 Nov 23;66(2):255–66. doi: 10.1007/s00125-022-05833-z (PMC9685151; doi:10.1007/s00125-022-05833-z)
Supplement: Supplementary file 1 — (PPTX 212 kb) [file 125_2022_5833_MOESM1_ESM.pptx]

## Slide 1
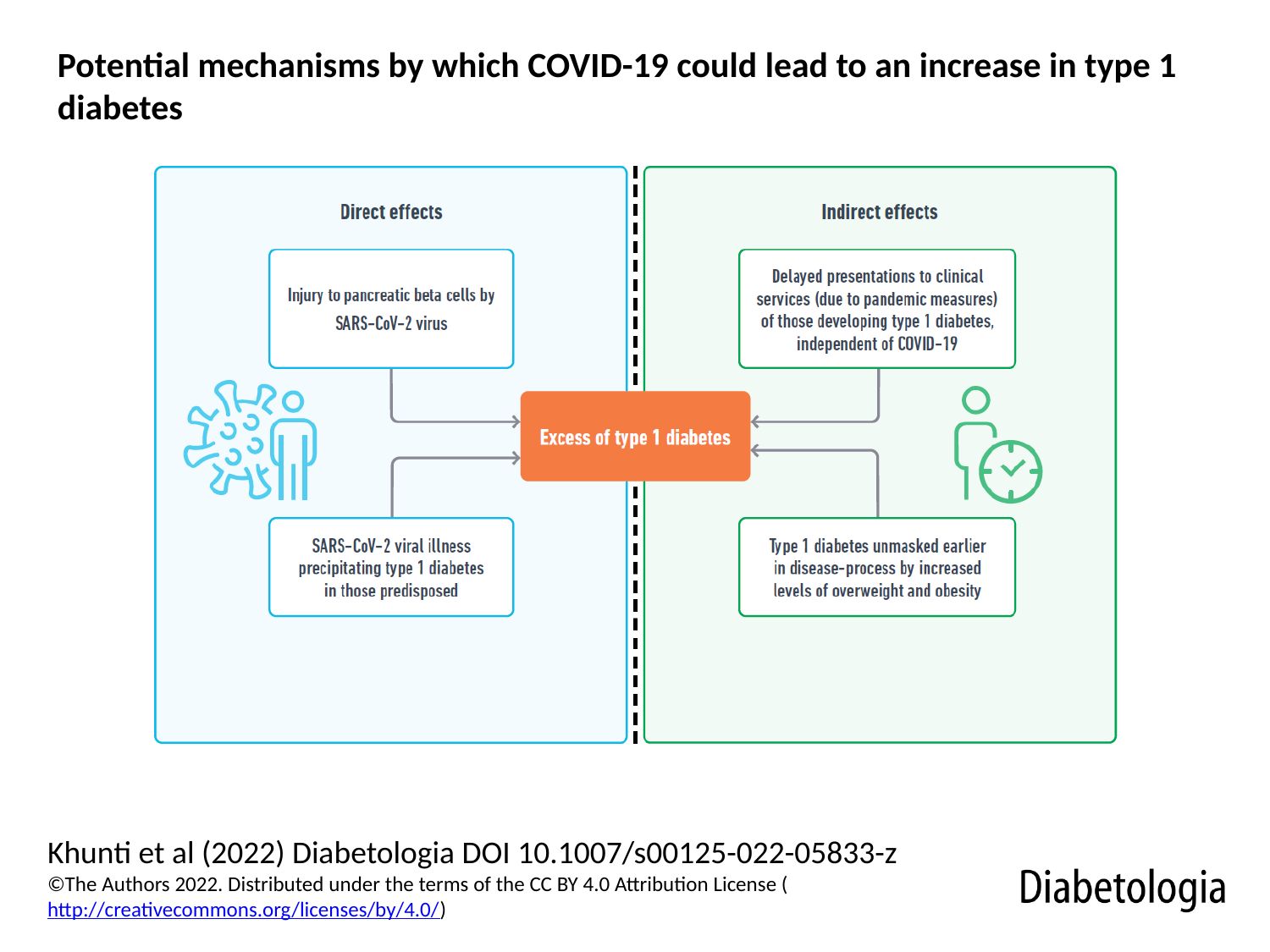

Potential mechanisms by which COVID-19 could lead to an increase in type 1 diabetes
Khunti et al (2022) Diabetologia DOI 10.1007/s00125-022-05833-z
©The Authors 2022. Distributed under the terms of the CC BY 4.0 Attribution License (http://creativecommons.org/licenses/by/4.0/)

## Slide 2
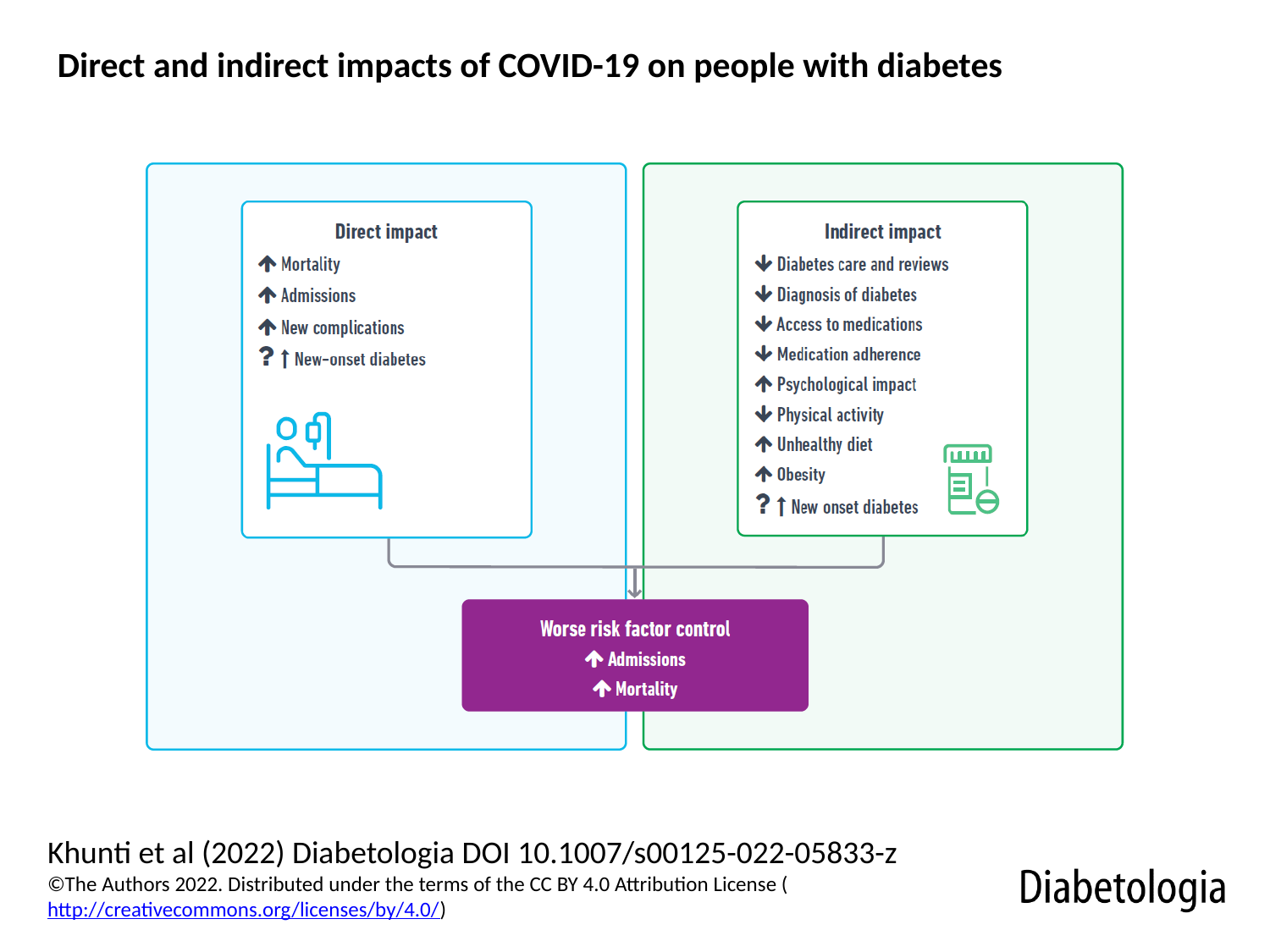

Direct and indirect impacts of COVID-19 on people with diabetes
Khunti et al (2022) Diabetologia DOI 10.1007/s00125-022-05833-z
©The Authors 2022. Distributed under the terms of the CC BY 4.0 Attribution License (http://creativecommons.org/licenses/by/4.0/)
